# Seasonal influenza: Knowledge, attitude and vaccine uptake among adults with chronic conditions in Italy

**DOI:** 10.1371/journal.pone.0215978

**Published:** 2019-05-01

**Authors:** Gaia Bertoldo, Annalisa Pesce, Angela Pepe, Concetta Paola Pelullo, Gabriella Di Giuseppe, The Collaborative Working Group

**Affiliations:** Department of Experimental Medicine, University of Campania “Luigi Vanvitelli”, Naples (Italy); Universita degli Studi di Ferrara, ITALY

## Abstract

This cross-sectional study aimed at evaluating the knowledge and attitudes concerning influenza vaccination in Southern Italy, and investigating the potential determinants of vaccine uptake. The sample consisted of 700 adults (mean age 58.7y) with chronic diseases attending four public specialty clinics in Italy. Overall, 64.7% of the participants were aware that influenza can be prevented with vaccines and that patients with chronic diseases are at higher risk of developing severe complications. Less than half of the sample (42.1%) received influenza vaccine in the last season, and 46.9% declared the will to receive influenza vaccination in the next season. The level of awareness was significantly lower among the elderly (> = 65y) and those with a higher self-reported health. A significantly higher likelihood of vaccination was observed among the elderly, the subjects with a higher knowledge about vaccine utility and safety, the participants with chronic respiratory diseases, and those who had taken more drugs. Future education programs and communication strategies are strongly needed in adults with chronic diseases to improve influenza vaccination knowledge and uptake.

## Introduction

It is well known that seasonal influenza is an acute respiratory illness caused by virus strains that annually undergo antigenic variations. Influenza is a major public health issue, having been associated with increased in all-cause mortality [1–2], significant economic costs due to work absenteeism and intensified healthcare service use [3]. The World Health Organization (WHO) estimates that annual influenza epidemics result in about 3 to 5 million cases of severe illness worldwide each year, and about 290.000 to 650.000 deaths [4]. The European Centre for Disease Prevention and Control estimates that up to 40,000 people in the European Union die prematurely each year due to causes associated with influenza [5].

Influenza surveillance is carried out in most developed countries by national networks of physicians who traditionally report cases from individuals with influenza-like illness (ILI) [6]. This is done to estimate the weekly incidence of influenza cases in winter season to determine the duration and intensity of the epidemic. In Italy, the sentinel surveillance system for ILI is called INFLUNET and is coordinated by the Italian National Institute of Health in collaboration with Centro Interuniversitario per la Ricerca sull’Influenza in Genova with the support of the Ministry of Health [7]. In Italy, ILI affects between 4% and 12% of the Italian population every year [8]. During the 2017/18 season, there were approssimately 8.677.000 medically attended ILI cases in Italy [9]. A surveillance system has been established to deal with severe and complicated laboratory-confirmed cases of influenza [10]. Every Italian region must report all laboratory-confirmed influenza cases (complicated or severe) that require intensive care unit hospitalisation, to the Ministry of Health and the National Health Institute [11]. The resulting data show that in the 2017/18 season, 764 severe laboratory-confirmed hospitalised cases were registered of which 173 resulted in death. Eighty-four percent of these severe cases, and 92.5% of the deaths surveyed showed the presence of at least one pre-existing chronic disease for which the influenza vaccine is recommended [12].

Annual vaccination has been demonstrated to be effective in reducing influenza-associated morbidity and mortality [4,13], especially in elderly individuals [14–15], and in reduction costs among high-risk patients [16–18]. There is good international evidence that some groups of people with chronic conditions are at higher risk of having adverse outcomes from influenza infection and would therefore benefit from vaccination [4,13,19–21]. Influenza-related complications in these groups include acute myocardial infarction and cardiovascular death, primary viral and secondary bacterial pneumonia, renal failure, and neurological syndromes [22–23]. The chronic medical conditions for which influenza vaccination is recommended includes diseases as diabetes, chronic cardiovascular or respiratory diseases, renal failure and immuno deficiency disorders [13,19–20].

Despite influenza’s severity and the availability of safe vaccines and policy recommendations, influenza vaccine uptake rates regularly fail to reach the recommended vaccination coverage target of 75% set by national and international programs within specific risk groups [21–24].

In the literature, several studies have investigated factors associated with influenza vaccine uptake, but most of them address the general population [25–27] or focus on specific risk groups [28–36]. Few investigations regarding vaccine uptake have been conducted among adults with underlying chronic conditions [37–38]. Therefore, it seemed interesting to conduct a survey to achieve the following primary objectives: 1) to assess the knowledge and attitudes about seasonal influenza and relative vaccine among adults with underlying chronic conditions, 2) to estimate the influenza vaccine uptake and 3) to identify the determinants associated with these outcomes.

## Materials and methods

### Study setting and population

We conducted a cross-sectional survey, from March to June 2017 and from February to April 2018 among adults with chronic diseases attending public specialty clinics in the city of Naples, Italy. Patients included in the analysis were required to be aged 18 years and over and to have at least one of the following three chronic medical conditions: diabetes (types 1 and 2), chronic respiratory disease and cardiovascular disease. A participant was defined as chronically ill if he/she had been diagnosed to have any of the above mentioned diseases by a physician during his/her lifetime.

The sample was selected with a two stages cluster sampling strategy. In the first stage, four hospitals (three general hospitals and one teaching hospital) were randomly selected from a list of all hospitals in Naples. Then, in each public outpatient clinic, a random sample technique was used in recruiting patients.

The minimum sample size required for the study was estimated in 374 patients, assuming expected influenza vaccination coverage rate of 42% [21,31], confidence interval of 95% and 5% margin of error. The minimum sample size was calculated as follows.

$$n=\frac{Z\frac{\alpha 2}{2}P(1-P)}{d^{2}}$$

To ensure that the sample was representative of the population, we considered a non-response rate of 30%. Therefore, the final sample size was 534 patients.

### Procedure

The directors of the selected public health centres received letters inviting them to participate in the survey.

Eligible patients attending the selected outpatient clinics (i.e. diabetology, cardiology, pulmonology) were asked to grant an anonymous, face-to-face interviews, by one of three trained preventive medicine residents.

### Instrument

The pretested structured questionnaire was completed after verbal consent was obtained from the participants. The questionnaire (S1 and S2 Files) comprised the following five sections: (1) socio-demographic characteristics (age, sex, nationality, educational level, marital status, number of children, number of cohabiting, employment status) and anamnestic characteristics (duration of disease, history of other comorbid conditions, pharmacological treatment prescribed, Morisky medication adherence, smoking status, alcohol consumption, number of physician contacts in the past 12 months, self-reported health status); (2) knowledge about vaccines, influenza and its vaccination (vaccine-preventable disease, groups at high risk of serious flu complications); (3) attitude towards influenza and its vaccination (concern about contracting influenza, usefulness of the vaccine, safety of the vaccine) were measured with a 10-point Likert scale ranging from 1 to 10; for assessing perceived severity, frequency and preventability of influenza, response options included ‘agree’, ‘disagree’, or ‘unsure’; for assessing intention to be vaccinated in the future, response options included ‘yes’ and ‘no’, and for each response patients were asked for their reasons; (4) about behaviors regarding influenza vaccination in the last five years, pneumococcal and shingles vaccination (whether or not they had received vaccination), response options included ‘yes’ and ‘no’, and for each response patients were asked for their reasons; (5) sources of information about influenza and its vaccination.

To assess patients’ self-reported medication adherence, we used the four item Morisky Medication Adherence Scale [39]. This scale, measures medication adherence in patients with chronic diseases over the previous month through four yes/no questions. The total score ranges from 0 (high adherence) to 4 (low adherence).

A pilot study was conducted with a sample of 50 patients (included in the final sample) to determine the comprehensibility of each question. The protocol was presented and approved by the Ethics Committee of the University of Campania “Luigi Vanvitelli”.

### Statistical analysis

In this study there were four outcomes of interest: a) knowledge that influenza is preventable by vaccine and that patients with chronic disease are at higher risk of developing severe forms of influenza (no = 0; yes = 1) (Model 1); b) positive attitude towards the utility of influenza vaccination (≤5 = 0; ≥6 = 1) (Model 2); c) receipt of influenza vaccination in the last season (no = 0; yes = 1) (Model 3); d) willingness to receive influenza vaccination in the next season (no = 0; yes = 1) (Model 4). The following independent variables were included in all models: age (three categories: 18–49 years; 50–64 years; ≥65 years), gender (male = 0; female = 1), marital status (other = 0, married = 1), occupation (unemployed = 0, employed = 1), education level (three categories: middle school or lower = 1, high school = 2, college degree or higher = 3), number of sons (none = 0; 1 = ≥1), number of cohabiting (none = 0; 1 = ≥1), duration of chronic disease (≤4 years = 0; ≥5 years = 1), having cardiovascular disease (no = 0; yes = 1), having diabetes (no = 0; yes = 1), having chronic respiratory disease (no = 0; yes = 1), having another disease for which vaccination is recommended (no = 0; yes = 1), number of drugs taken (0 = ≤4;1 = ≥5), making at least one visit to physician in the last year (no = 0; yes = 1), Morisky medication adherence (low adherence = 0; high adherence = 1), perception of personal health status (poor (1–7) = 0; good (8–10) = 1), considering physicians as source of information about influenza vaccination (no = 0; yes = 1), need for additional information about influenza vaccination (no = 0; yes = 1). The variables knowledge that influenza is preventable by vaccine and that patients with chronic disease are at higher risk of developing severe forms of influenza (no = 0; yes = 1), and having heard about vaccination from physician (no = 0; yes = 1), were included in Models 2, 3 and 4. Finally, positive attitude towards the utility of influenza vaccination (low (1–5) = 0; high (6–10) = 1), perception of being at high risk of contracting influenza (low (1–5) = 0; high (6–10) = 1), and attitude towards the dangers of influenza vaccination (low (1–5) = 0; high (6–10) = 1), were included in Models 3 and 4.

Analysis (S3 File) were performed using the software Stata 15 [40]. Bivariate analysis was carried out to assess the association between each of the independent characteristics and the different outcomes of interest. After bivariate analyses, variables associated with the outcomes at the *p*-value ≤0.25 level were subsequently introduced into a multivariate regression model [41]. Then, stepwise logistic regressions analysis was performed to evaluate the associations between the independent characteristics and outcomes of interest. Interaction terms were tested in the multiple logistic regression models. The results of the multivariate analysis were expressed in odds ratios (ORs) and 95% confidence intervals (CIs), with a statistically significant level of *p*-value ≤0.05.

## Results

In total, 700 of the 712 subjects agreed to participate in the study, with an overall response rate of 98.3%. Table 1 shows an overview of the respondents’ socio-demographic characteristics. The mean age of the participants was 58.7 years (18–92 years). Slightly more than half were females, and almost all were Italian. A total of 38.5% had completed a high school education, about 60% were married and only 30.6% were employed. Moreover, patients were mainly affected by diabetes (48.7%), cardiovascular disease (40.4%) and chronic respiratory disease (36.3%).

**Table 1:** Socio-demographic informations and influenza vaccination uptake of the study population.

			*Influenza vaccination uptake in the previous season (n = 700)*				
			Yes		No		
	N	%	N	%	N	%	*p*
*Age (700)*	58.6±19.2 (18–92)*		64.8±16.9 (18–92)*		54.2±19.6 (18–89)*		
18–49	177	25.3	44	24.9	133	75.1	
50–64	173	24.7	55	31.8	118	68.2	<0.0001
≥65	350	50.0	196	56	154	44	
*Sex (700)*							
Male	318	45.4	141	44.3	177	55.7	0.283
Female	382	54.6	154	40.3	228	59.7	
*Nationality (700)*							
Italian	689	98.4	291	42.2	398	57.8	0.696
Other	11	1.6	4	36.4	7	63.6	
*Education level (699)*							
No formal education	11	1.6	4	36.4	7	63.6	
Elementary	130	18.6	54	41.5	76	58.5	
Middle school	192	27.5	80	41.7	112	58.3	0.553
High school	269	38.5	119	44.2	150	55.8	
College degree	97	13.9	37	38.1	60	61.9	
*Underlying chronic disease°*							
Diabetes	341	48.7	155	45.5	186	54.5	0.084
Cardiovascular disease	283	40.4	118	41.7	165	58.3	0.844
Chronic respiratory disease	254	36.3	123	48.4	131	51.6	0.011
*Employment status (699)*							
Not employed	485	69.4	148	36.5	256	63.2	<0.0001
Employed	214	30.6	66	22.4	229	77.6	
*Marital status (700)*							
Married	415	59.3	188	63.7	227	56.1	0.001
Other	285	40.7	107	36.3	178	43.9	
*Number of cohabiting (700)*							
None	606	86.6	48	16.3	46	11.4	0.60
≥1	94	13.4	247	83.7	359	88.6	
*Number of sons (699)*							
None	189	27	63	21.4	126	31.1	0.004
≥1	510	73	231	78.6	279	68.9	
*Number of drugs taken (699)*							
≤4	406	58.1	139	47.3	267	65.9	<0.0001
≥5	293	41.9	155	52.7	138	34.1	
*Perception of personal health status (693)*	6.27±2.1 (1–10)*		6.14±1.9 (1–10)*		6.4±2.1 (1–10)*		0.061

*Mean±Standard deviation (Range)

°Often more than one chronic disease is associated

### Health status and life habits

All the participants went to clinics for routine check-up visits, particularly, 29.4% for cardiovascular disease, 33% for chronic respiratory disease and 37.6% for diabetes. Nearly all participants took drugs. As regards alcohol use, 38.4% of the participants never drank alcohol, whereas 13.2% drank regularly. Regarding smoking, 60% had been smokers during their lifetime, and 41.1% were active smokers.

Meanwhile, 73.3% had at least a health checkup in the last year, 28.3% presented to the emergency department and 17% were admitted to hospitals. A total of 27% of the responders declared having good health status. Finally, according to the Morisky scale, 75.7% had high medication adherence.

### Knowledge of influenza vaccination

A total of 98.6% of the respondents reported that they had heard of vaccines as interventions that protect us from serious infectious diseases. Moreover, patients correctly identified influenza (94.7%), meningitis (81.5%), pneumonia (44.2%) and shingles (20.6%) as vaccine-preventable diseases. Meanwhile, other diseases such as heart attack (95.1%), common cold (76.8%), AIDS (69.7%), and hepatitis C (46.6%) were identified incorrectly as vaccine-preventable diseases. Among the respondents, 64.7% knew that influenza can be prevented with vaccines and that patients with chronic diseases are at higher risk of developing severe forms of influenza. The results of the multivariate logistic regression model showed that those who had a worse perception of personal health status (OR = 0.64; 95% CI 0.44–0.92), were more likely to know that influenza is preventable by vaccine and that patients with chronic diseases are at higher risk of developing severe forms of influenza. Also, patients aged 18–49 years (OR 0.6; 95% CI 0.41–0.9) compared with those aged ≥65 years were less likely to know that influenza is preventable by vaccine. Moreover, educational level was found to have an impact on knowledge, given that patients with a middle school or lower (OR = 0.38; 95% CI 0.21–0.67) and high school (OR = 0.42; 95% CI 0.24–0.75) education were less knowledgeable compared with those with a college degree or higher (Model 1 in Table 2).

**Table 2:** Multivariate logistic analysis to characterize factors associated with different outcomes of interest.

Variable	OR	SE	95% CI	*p* value
**Model 1.** Knowledge that influenza is preventable by vaccine and that patients with chronic disease are at higher risk of developing severe forms of influenza(no = 0; yes = 1)				
Log likelihood = -424.31, χ^2^ = 43.38 (7 df), *p*< 0.0001 (sample size = 689)				
Educational level				
Middle school or lower	0.38	0.11	0.21–0.67	0.001
High school	0.42	0.12	0.24–0.75	0.003
College degree or higher	1.0*	-	-	-
Age				
18–49	0.6	0.12	0.4–0.9	0.013
≥65	1.0*	-	-	-
Perception of personal health status (poor = 0; good = 1)	0.63	0.12	0.44–0.92	0.017
**Model 2**. Positive attitude towards the utility of influenza vaccination (no = 0; yes = 1)				
Log likelihood = -356.16, χ^2^ = 37.86 (10 df), *p*<0.001(sample size = 622)					
Information about influenza vaccine from physician (no = 0; yes = 1)	2.18	0.44	1.47–3.23	<0.0001
Educational level				
High school	1.59	0.32	1.07–2.37	0.021
College degree or higher	1.0*	-	-	-
**Model 3**. Had received influenza vaccination in the last season (no = 0; yes = 1)				
Log likelihood = -301.37, χ^2^ = 257.11 (11 df), *p*<0.001 (sample size = 628)				
Age				
18–49	0.21	0.74	0.11–0.42	<0.0001
50–64	0.3	0.72	0.18–0.48	<0.0001
≥65	1.0*	-	-	-
Information about influenza vaccine from physician (no = 0; yes = 1)	3.11	0.64	2.08–4.67	<0.0001
Awareness of usefulness of influenza vaccine (no = 0; yes = 1)	7.21	1.96	4.23–12.28	<0.0001
Attitude towards the dangers of influenza vaccination (negative = 0; positive = 1)	0.2	0.05	0.12–0.34	<0.0001
Patients with chronic respiratory diseases (no = 0; yes = 1)	1.72	0.37	1.13–2.61	0.011
Number of drugs taken (≤4 = 0; ≥5 = 1)	1.69	0.36	1.1–2.6	0.015
Knowledge that influenza is preventable by vaccine and that patients with chronic disease are at higher risk of developing severe forms of influenza (no = 0; yes = 1)	1.63	0.36	1.06–2.51	0.026
Have a longer length of disease (≤4 years = 0; ≥5 years = 1)	1.64	0.41	1.01–2.69	0.047
**Model 4.** Willingness to receive influenza vaccination in the next season (no = 0; yes = 1)				
Log likelihood = -287.43, χ^2^ = 294.64 (13df), p<0.001 (sample size = 628)				
Awareness of usefulness of influenza vaccine (no = 0; yes = 1)	9.97	2.78	5.77–17.2	<0.0001
Attitude towards the dangers of influenza vaccination (negative = 0;positive = 1)	0.13	0.04	0.08–0.24	<0.0001
Information about influenza vaccine from physician (no = 0; yes = 1)	2.89	0.63	1.88–4.44	<0.0001
Age				
18–49	0.31	0.1	0.16–0.62	0.001
50–64	0.29	0.07	0.18–0.48	<0.0001
≥65	1.0*	-	-	-
Knowledge that influenza is preventable by vaccine and that patients with chronic disease are at higher risk of developing severe forms of influenza (no = 0; yes = 1)	1.94	0.43	1.25–3.01	0.003
Perception of risk of contracting influenza (low = 0; high = 1)	1.92	0.04	1.2–3.1	0.007
Patients with chronic respiratory diseases (no = 0; yes = 1)	1.79	0.39	1.15–2.77	0.009
Number of drugs taken (≤4 = 0; ≥5 = 1)	1.74	0.44	1.05–2.87	0.031

*Reference category in multivariate analysis

### Attitudes towards of influenza vaccination

Regarding perceived severity, 5.3% and 17.9% believed that influenza was rare and serious, respectively, and 64.3% agreed that influenza may be prevented. The mean value of the perceived personal risk of contracting influenza was 3.9 on a scale of 1 to 10, with values ≥6 indicating a perception of being at risk. Patients were asked about their beliefs towards the dangers of influenza vaccination and 25.6% considered it very unsafe, with a mean total score of 3.9.

Patients’ attitudes regarding the usefulness of influenza vaccine were measured on a 10-point Likert scale ranging from 1 to 10 with higher scores indicating higher utility The mean total value was 7 and 71.6% expressed very favourable attitude by giving scores ranging from 6 to 10 towards the usefulness of influenza vaccine. The multivariate logistic regression showed that patients with high school educational level compared with those with a college degree or higher (OR = 1.59; 95% CI 1.07–2.37) and those who had received information about influenza vaccine from physicians (OR = 2.18; 95% CI 1.47–3.23), were more likely to think that the influenza vaccine is very useful (Model 2 in Table 2).

### Influenza vaccination uptake

With regard to behaviors, 42.1% had received influenza vaccine in the last season, of which 33.6% are aged under 64 years and 66.4% were older than 64 years (Table 3). Moreover, 39.4% of the patients had received influenza vaccine in the five previous years. Meanwhile, 52.5%, 41.7% and 40% of the patients affected by diabetes, chronic respiratory disease and cardiovascular disease had received influenza vaccine. Mainly, the influenza vaccine was recommended by physicians (74.9%), and only 6.4% reported receiving recommendations from specialists. Among those who indicated reasons for not being vaccinated, the main reasons were fear of adverse effects (24.9%), lack of recommendation from physician (24.2%), beliefs that they were not at risk for influenza (18.3%) and the notion that the vaccine is not useful (14%).

**Table 3:** History of influenza vaccination and willingness to receive it.

	N	%
Vaccinated in the previous 5 years*		
No	169	60.6
Yes	110	39.4
*Vaccinated in the last season*		
No	405	57.9
Yes	295	42.1
*Vaccination was recommended by°*		
Physicians	221	74.9
Specialists	19	6.4
Other	55	18.7
*Main reasons for not receiving influenza vaccination*^*+*^		
Fear of adverse effects	101	24.9
It has not been recommended by physician	98	24.2
I do not feel at risk	74	18.3
Influenza vaccine is not useful	57	14
*Willingness to receive influenza vaccination*		
No	372	53.1
Yes	328	46.9
*Main reasons for unwillingness to receive influenza vaccination*^*§*^		
Fear of adverse effects	90	41.5
I do not feel at risk	62	28.6
Influenza vaccine is not useful	41	18.9
*Main reasons for willingness to receive influenza vaccination*^*#*^		
Influenza vaccine is useful	252	76.8
If recommended by physician	41	12.5
I feel at risk	32	9.7

* Only for those with a diagnosis of chronic disease by more than 5 years

° Only for those vaccinated in the last season

^+^ Only for those not vaccinated in the last season

^*§*^ Only for those who would receive influenza vaccination in future

^#^ Only for those who would not receive influenza vaccination in future

Those who considered influenza vaccine useful (OR = 7.2; 95% CI 4.23–12.28), those who did not think that influenza vaccine is dangerous (OR = 0.2; 95% CI 0.12–0.34), those who had taken more drugs (OR = 1.69; 95% CI 1.11–2.58), those who had received information about influenza vaccine from physicians (OR = 3.11; 95% CI 2.08–4.67), those who had a longer length of disease (OR = 1.64; 95% CI 1.01–2.69), those with chronic respiratory diseases (OR = 1.72; 95% CI 1.13–2.61) and those who knew that influenza is preventable by vaccine and that patients with chronic diseases are at higher risk of developing severe forms of influenza (OR = 1.63; 95% CI 1.06–2.51) were more likely to receive influenza vaccine in the last season (Model 3 in Table 2). Moreover, patients aged 18–49 years (OR = 0.21; 95% CI 0.11–0.42) and 50–64 years (OR = 0.3; 95% CI 0.18–0.48) compared with patients aged ≥65 years were less likely to receive influenza vaccine in the last season.

### Willingness to receive influenza vaccination in the next season

Among those that had not received influenza vaccine in the last season, only 11.6% were not willing to receive influenza vaccine in future.

Furthermore, as regards the willingness to receive influenza vaccine, only less than half (46.9%) responded ‘yes’ when asked whether they were willing to receive influenza vaccine (Table 3). The results of the multivariate analysis showed that those who were more willing to receive influenza vaccine were those who considered the influenza vaccine useful (OR = 9.97; 95% CI 5.77–17.22), those who did not considered the influenza vaccine to be dangerous (OR = 0.14; 95% CI 0.08–0.24), those who perceived being at risk of contracting influenza (OR = 1.92; 95% CI 1.19–3.11), those who had received information about influenza vaccine from physicians (OR = 2.89; 95% CI 1.88–4.44), those who had a longer length of disease (OR = 1.74; 95% CI 1.05–2.87), those with chronic respiratory disease (OR = 1.79; 95% CI 1.16–2.77) and those who knew that influenza is preventable by vaccine and that patients with chronic disease are at higher risk of developing severe forms of influenza (OR = 1.94; 95% CI 1.25–3.01). Moreover, patients who were aged 18–49 years (OR = 0.31; 95% CI 0.16–0.62) and 50–64 years (OR = 0.29; 95% CI 0.18–0.48) compared with patients aged ≥65 years, were less willing to receive influenza vaccine (Model 4 in Table 2).

When the willingness to receive influenza vaccine was investigated, the main reason were belief that the vaccine is useful (76.8%), if recommended by physician (12.5%), and feeling at risk (9.7%).

### Sources of information

As regards sources of information about influenza vaccine, the most frequently mentioned were physicians (38.3%) and television/newspapers (32.3%); 55.9% indicated that they would like more information about influenza vaccine.

## Discussion

Annual influenza vaccination has been demonstrated to be effective in reducing morbidity and mortality among high-risk patients. The present study collected detailed data on the knowledge, attitudes and vaccine uptake among a sample of adults with chronic diseases. The current study’ findings can be compared with those in other studies despite the latter’s different samples and methodologies.

Regarding the knowledge of the respondents, the current study revealed that only 64.7% knew that influenza can be prevented by vaccination and that patients with chronic diseases are more likely to develop severe forms of influenza. This finding was congruent with that of another study conducted in Italy, where 64.2% of pregnant women correctly answered that influenza is more dangerous for pregnant women than for women who are not pregnant [35]. By contrast, a considerably lower level of knowledge was observed in a study performed in the United States (US) among the general population, wherein only 19.6% of the respondents indicated correct knowledge of the influenza vaccination recommendation [25], and in a study conducted in France on a sample of patients at high-risk for severe influenza who arrived at the emergency department [38]. Meanwhile, a higher level of knowledge has been observed in other studies with different samples. In Australia, 85.9% of healthcare workers knew that they were recommended for seasonal influenza vaccination [25]. In our multivariate analysis, having a higher education was significantly associated with a higher level of knowledge about influenza and its vaccination. As in the present study, this characteristic was found to be an associated factor in a previously cited study [19]. These findings are important for identifying intervention programs to improve knowledge about influenza vaccination recommendation among groups with lower educational levels. In addition, reporting having received informations about vaccination from physicians was also significantly associated with a higher level of knowledge, but this finding did not reach statistical significance in the multivariate analysis. The fact that most of the participants did not know that influenza vaccination is recommended for them and mentioned physicians as their main source of information about influenza and its vaccination, underlines the importance of physicians in assuming the role of educators, given that adequate knowledge is a basis for adopting appropriate attitudes and practices.

Another main finding from this study was that only 17.9% of the sample considered influenza to be serious illness, and only 26.3% reported that they perceive themselves at risk of contracting influenza with a mean value of 3.9 on a scale from 1 to 10. This perceived susceptibility was lower than that observed in France [38]. Similarly, in another nationwide cross-sectional survey conducted in Germany, the median value of the participants’ perceived susceptibility and severity of influenza was 5 [30]. Furthermore, our results revealed a mean value of 3.9 towards the dangers of influenza vaccination. This finding was higher than that found in the two aforementioned studies from Germany and France [30,38].

It is interesting to note that 71.6% of the participants had a very positive attitude towards the usefulness of the influenza vaccine. This result is consistent with that of Casalino et al. [38]. Nevertheless, only 46.9% of the respondents intended to be vaccinated next season. This result is very important because it highlights the need to improve policy interventions to increase adherence to influenza vaccination among these high-risk individuals.

With regard to vaccination status, this study identified an uptake rate of 42.1% in the participants in the last season. This result revealed that more than half of those with chronic diseases (57.9%) were not immunised against influenza therefore, vaccination coverage remains far below the target of 75% [21]. Our result is similar to that reported in two studies conducted in Italy in 2012–13 and in Australia in 2008, although referred to a general population [32,37]. Most countries of the WHO European Region with influenza vaccine recommendations reported vaccine uptake rates below 40% [24] but much lower rates have been reported in studies conducted in France (32.5%) [38], Ireland (29.1%) [33] and Germany (23%) [30].

In our sample, the frequently reported reasons for not having had the vaccine were fear of side effects (24.9%), lack of physician recommendation (24.2%), belief of not being at risk for influenza (18.3%) and belief in the usefulness of the vaccine (14%). Our analysis showed that older age, informations about the influenza vaccine obtained through consultation with a physician, positive attitude towards the usefulness of influenza vaccination and the intention to be vaccinated next season, were predictors of vaccination status. Other studies showed similar results. Being aged 65 and over was found positively associated with vaccination uptake in Australia [37] and in Italy [27,32]. Likewise, physician recommendation was among the associated factors in two studies [33,38]. Having a positive attitude towards the usefulness of influenza vaccination was found to be significantly associated with vaccine uptake in the US, Italy and France [26,35,38]. In addition to our findings, a combination of several factors may affect influenza vaccine uptake rates. In particular, disease prevention policies in Italy are organised through the National Prevention Plan, which establishes that each Italian region should evolve its own Regional Prevention Plan. These regions should develop personalised projects based on their regional epidemiological contexts [42] and implement their own Regional Immunization Plans and schedules on the basis of this document. However, in a few regions, such as Campania Region, the financial deficit due to the decentralisation of healthcare from the central government to the regions, and the related fiscal federalism, has led to a redistribution of resources for public healthcare. Several studies have shown an association between local financial deficits and the low quality of prevention projects included in Regional Prevention Plans [42–44].

Our analysis showed that physicians’ advice plays an important role in the vaccination decision-making process among patients with chronic diseases. It is worth noting that in our study, influenza vaccine was more often recommended by general practitioners than by specialists. Nevertheless, all healthcare providers should play a key role in raising awareness and knowledge about the risk of developing complicated or severe influenza illnesses among individuals with underlying chronic conditions and hence, in increasing vaccination uptake. Indeed, it is reasonable that an endorsement from a trusted healthcare provider would be likely to result in greater adherence to influenza vaccination.

For appreciate the findings of the current survey to be appreciated, some potential limitations in the design and measurements need to be addressed. First, this survey adopted a cross-sectional research design and thus it did not permit analysis of the direction of influence between the different variables and the outcomes of interest. Second, there was potential bias attributable to the use of a self-reporting instrument, and we were unable to accurately determine vaccine uptake. Third, we have considered a sample of people attending public outpatient clinic and this may have affected the estimate of vaccine uptake. Finally, the data were obtained from interviews and the answers were not verified through chart review. Therefore, it could not be ascertained whether the respondents answered correctly, and recall bias could have occurred. Despite these limitations, the major strength of the study was the high response rate, which made the results representative enough of the population.

In conclusion, our study results indicated a good level of knowledge about influenza, and positive attitudes towards influenza vaccine, but suboptimal seasonal vaccination coverage in Italian adults with underlying chronic diseases. Communication and awareness of influenza and its vaccine in this population is an important starting point, and all healthcare professionals and public health workers can play a key role in this regard. Future studies should be focused on implementing educational interventions for adults with chronic diseases by both general practitioners and specialists. Moreover, there is need for a different government approach to resolving the financial deficit in Italy focused on health promotion and disease prevention [43].

## Supporting information

S1 File S1_English questionnaire. (DOCX)

S2 File S2_Italian questionnaire. (DOCX)

S3 File Minimal dataset. (XLSX)
