# Supplementary material for: Seasonal influenza: Knowledge, attitude and vaccine uptake among adults with chronic conditions in Italy
Source: PLoS One. 2019 May 1;14(5):e0215978. doi: 10.1371/journal.pone.0215978 (PMC6493755; doi:10.1371/journal.pone.0215978)
Supplement: S1 File — (DOCX) [file pone.0215978.s001.docx]

**QUESTIONNAIRE**

**Section A.**

**This section is designed to gather information about your socio-demographic characteristics.**

**A1.** Sex: **□** Male **□** Female

**A2.** How old were you on your last birthday? _______

**A3.** What is your weight and stature?____________

**A4.** What is your nationality?_____________

**A5.** What is your highest level of education?_________________________________________________

**A6.** What is your marital status? □ married □ single (never married) □ other ___________

**A7.** What is your occupation?_____________________________________________________________

**A8**. How many people live with you?________

**A9.** How many sons do you have?

**Section B.**

**This section is designed to gather information about your health status, lifestyle habits and need for medical visits.**

**B1.** Why did you go to outpatient clinic today? _____________________________________

**B2.** Do you have any diseases and for each one how long have you suffered? **__________________________**

**B3.** Do you take drugs? □ no □ yes, what?____________

**B4.** Have you smoked at least 100 cigarettes in all your life? (5 packs of 20 cigarettes) □ no □ yes **(go to the question B5.)**

**B5.** Do you currently smoke cigarettes? □ yes, how many?______□ no □ no I stopped, because,_________)

**B6.** Have you been to a physician in the last year? □ no □ yes, (how many times?________)

**B7.** Have you been to a specialist physician in the last year? □ no □ yes, (how many times?________)

**B8.** Have you been to the emergency room in the last year? □ no □ yes, (how many times?________), why? ___________

1) _______________________________________ 2) ______________________________________

3) _______________________________________ 4) ______________________________________

**B9.** Have you been hospitalized in the last year? □ no □ yes, (how many times?________), why? _______

1) _______________________________________ 2) ______________________________________

3) _______________________________________ 4) ______________________________________

**B10.** On a scale from 1 to 10, how would you classify your current state of health? (1 bad; 10 very good)______

**Section C. The questions in this section aim at gathering informations about your medication-taking behavior.**

**Morisky scale** (Yes =1, No=0; Range: 0-4; score 0-1= high adherence /score 2-4= low adherence)

Over the last month, did you ever forget to take your medicines? □ no □ yes

Are you careless at times about taking your medicines? □ no □ yes

Over the last month, did you sometimes stop taking your drugs when you felt better ? □ no □ yes

Over the last month, did you sometimes stop taking your drugs if you felt worse after taking them?

□ no □ yes

**Section D.**

**This section is designed to gather information about your knowledge of vaccine-preventable diseases**

**D1.** Vaccinations are interventions that protect against infectious disease, have you ever heard of them? (more than one answer is possible) □ no □ yes, from whom? ____________________________________________

**D2.** Indicate, between following diseases, those that can be avoided with vaccination?

□ influenza □ [pneumonia](https://www.cdc.gov/pneumonia/index.html) □ meningitis □ shingles □ AIDS /HIV □ hepatitis C □ heart attack □ cold

**D3.** In your opinion, which of the following subjects are at higher risk of developing severe forms of influenza?

□ children <6 months old □ children and young persons (5-18 years) □<64 years old with chronic conditions □ healthy young adults □ elderly ( ≥65 years old) □ pregnant women □ other____________________

| **D4.** For each of the following statements indicate whether you are in agreement, uncertain or disagree | **agreement** | **uncertain** | **disagree** |
| --- | --- | --- | --- |
| Influenza is rare | □ | □ | □ |
| Influenza is serious | □ | □ | □ |
| Influenza may be prevented | □ | □ | □ |

**Section E.**

**This section is designed to gather information about your attitudes towards influenza and influenza vaccinations.**

**E1.** On a scale from 1 to 10, how much are you worried about developing influenza? (1 not worried; 10 very worried)________

**E2.** On a scale from 1 to 10, how useful do you believe vaccination to prevent influenza? (1 not useful; 10 very useful) ________

**E3.** On a scale from 1 to 10, how dangerous do you believe influenza vaccination to be? (1 not dangerous; 10 very dangerous) ________

**Section F. Behaviors.** In this section I will ask you some questions on health-related behaviors.

**F1.** Did you receive a vaccination against influenza in in the last season?

□Who advise you to have it?: ____________________________________________________

□If no, why? _________________________________________________________________

**F2.** Did you receive a vaccination against influenza in the last 5 years?

|  | **YES** | **NO** | **NOT SURE** | **REASON** |
| --- | --- | --- | --- | --- |
| 2015 |  |  |  |  |
| 2014 |  |  |  |  |
| 2013 |  |  |  |  |
| 2012 |  |  |  |  |

**F3.** During the last 12 months, did you receive any of these vaccinations?

|  | **YES** | **NO** | **NOT SURE** | **REASON** |
| --- | --- | --- | --- | --- |
| Meningococcus |  |  |  |  |
| Pneumococcus |  |  |  |  |
| Shingles |  |  |  |  |
| Other |  | | | |

**F4.** Do you intend to receive a vaccination against influenza next winter?

□ if yes, why?____________________________________________________________________

□if no, why?______________________________________________________________________

□if not sure, why? _________________________________________________________________

**F5.** Did any of your household members (wife/husband, children, caregiver, etc…) receive a personal invitation from a physician for the influenza vaccination?

□not sure □no □yes, they received flu vaccine last winter □yes, they received flu vaccine during the last 5 years.

**F6.** In your opinion, how much will it cost you the influenza vaccination?

□ it is totally at my charge □ it is totally free □ it is partially at my charge

**□** it is at my charge with a partial reimburse by private/company insurance policies **□** other__________

**Section G.**

**This section is designed to assess the sources from which you acquire information about influenza vaccination**

**G1.** Have you received information about influenza vaccination? (more than one answer is possible)

□ no □ yes, from whom? ___________________________________________________

**G2.** How useful do you believe information about influenza vaccination to be?

□ very bad □ bad □ moderate □ good □ very good

**G3.** Do you feel you need more information about influenza vaccination? □ no □ yes
