# Supplementary material for: Seasonal influenza: Knowledge, attitude and vaccine uptake among adults with chronic conditions in Italy
Source: PLoS One. 2019 May 1;14(5):e0215978. doi: 10.1371/journal.pone.0215978 (PMC6493755; doi:10.1371/journal.pone.0215978)
Supplement: S2 File — (DOCX) [file pone.0215978.s002.docx]

**QUESTIONARIO**

**Sezione A.** In questa sezione Le farò domande relative ad alcune caratteristiche socio-anagrafiche.

**A1.** Sesso □ M □ F

**A2.** Quanti anni ha? ________

**A3.** Qual è la sua nazionalità? □ Italiana □ altro**____________________________________**

**A4**. Qual è il più elevato titolo di studio che ha conseguito?

□ nessuno □ elementare □ media-inferiore □ media-superiore □ laurea □ altro

**A5.** Qual è il Suo stato civile? □ celibe/nubile □ sposato/a □ altro

**A6.** Qual è la Sua attuale attività lavorativa? ______________

**A7.** Escluso Lei, con quante persone vive? ________

**A8.** Ha figli? □ no □ sì, quanti? ____

**Sezione B.**

Le domande che seguono servono a rilevare il suo stato di salute, le abitudini di vita e il suo bisogno di visite mediche.

**B1.** Per quale motivo oggi si è recato in ambulatorio? _____________________________________

**B2.** Può dirmi le malattie di cui soffre e per ciascuna specificare da quanto tempo ne soffre?

**__________________________________________________________________________________**

**__________________________________________________________________________________**

**B3.** Assume farmaci? □ no □ sì, quali ____________________________

**B4.** In tutta la Sua vita ha fumato almeno 100 sigarette (5 pacchetti da 20 sigarette)? □ no □ si **(andare alla B5.)**

**B5.** Attualmente fuma sigarette? □ si, quante al giorno______ □ no □ no ho smesso, perché,_________ )

**B6.** Negli ultimi 12 mesi si è rivolto/a al suo medico di medicina generale? no si, quante volte ? _______

**B7.** Negli ultimi 12 mesi si è rivolto/a medici specialisti? no si, quante volte ? _____________

**B8.** Negli ultimi 12 mesi si è recato/a al Pronto Soccorso? no si, quante volte? ___________, per quale motivo?

1) _______________________________________ 2) ______________________________________

3) _______________________________________ 4) ______________________________________

**B9.** Negli ultimi 12 mesi è stato ricoverato in Ospedale?

no si, quante volte? ________, per quale motivo?

1) _______________________________________ 2) ______________________________________

3) _______________________________________ 4) ______________________________________

**B10.** In una scala da 1 a 10 come giudica il Suo stato di salute attuale? **(1 indica cattivo e 10 ottimo) _______**

**Sezione C.** Le domande in questa sezione sono mirate a rilevare informazioni riguardo le modalità con cui segue le terapie.

**Scala di Morisky per l’aderenza** (Si=1, No=0; punteggio 0-1 alta aderenza - punteggio 2-4 bassa aderenza)

Nell’ultimo mese, si è mai dimenticato/a di assumere farmaci? no si

A volte è poco attento alle sue terapie (es. orari)? no si

Nell’ultimo mese, quando si è sentito meglio a volte ha interrotto la terapia? no si

Nell’ultimo mese, quando si è sentito peggio a volte ha interrotto la terapia? no si

**Sezione D. Conoscenze** In questa sezione Le farò alcune domande sulle sue conoscenze in tema di vaccini.

**D1.** Le vaccinazioni sono interventi che ci proteggono da gravi malattie infettive.

Ne ha mai sentito parlare? □no □sì, da chi? ____________________________________________

**D2.** Secondo lei, quali delle seguenti malattie si possono evitare con la vaccinazione?

□ influenza □ polmonite □ meningite □fuoco di Sant’Antonio (Herpes zoster)

□ AIDS /HIV □ Epatite C □ infarto □ raffreddore comune

**D3.** Secondo lei, chi è più a rischio di sviluppare forme gravi di influenza?

□ bambini <6 mesi □ bambini/ragazzi 5aa-18aa □ <64 anni con malattie croniche

□ soggetti sani giovani □ anziani ( ≥65 anni) □ donne in gravidanza □ altro_____________

| **D4.** Per ciascuna delle seguenti affermazioni mi dica se è in accordo, incerto o in disaccordo | **d’accordo** | **incerto** | **disaccordo** |
| --- | --- | --- | --- |
| L’influenza è una malattia rara | □ | □ | □ |
| L’influenza è una malattia grave | □ | □ | □ |
| L’influenza è una malattia prevenibile | □ | □ | □ |

**Sezione E. Attitudini**

In questa sezione Le farò alcune domande su ciò che pensa sull’influenza e sulla vaccinazione antinfluenzale.

**E1.** In una scala da 1 a 10 quanta paura ha di contrarre l’influenza? **(1 indica nessuna paura e 10 molta paura) _________**

**E2.** In una scala da 1 a 10 quanto ritiene utile il vaccino per prevenire l’influenza?

**(1indica nessuna utilità e 10 molta utilità) _______**

**E3.** In una scala da 1 a 10 quanto ritiene pericoloso per la sua salute il vaccino antinfluenzale?

**(indicare con 1 nessun pericolo e con 10 molto pericoloso) _______**

**Sezione F. Comportamenti** In questa sezione Le farò alcune domande sui suoi comportamenti relativi alla salute.

**F1.** Si è vaccinato contro l’influenza lo scorso autunno-inverno (Ottobre-Dicembre 2017)?

sì, da chi le è stato consigliato?: ________________________________________________________

no, perché? _______________________________________________________________________

**F2.** Si è vaccinato contro l’influenza negli ultimi 5 anni? (solo se la diagnosi > 5 anni)

|  | **SÌ** | **NO** | **NON RICORDO** | **MOTIVO** |
| --- | --- | --- | --- | --- |
| 2016 |  |  |  |  |
| 2015 |  |  |  |  |
| 2014 |  |  |  |  |
| 2013 |  |  |  |  |

**F3.** Nell’ultimo anno ha fatto qualcuna delle seguenti vaccinazioni?

|  | **SÌ** | **NO** | **NON RICORDO** | **MOTIVO** |
| --- | --- | --- | --- | --- |
| Pneumococco |  |  |  |  |
| Zoster |  |  |  |  |
| Altro |  | | | |

**F4.** Pensa di vaccinarsi il prossimo anno contro l’influenza?

sì, perché?___________________________________________________________________

no, perché? __________________________________________________________________

non so, perché? _______________________________________________________________

**F5.** Ai suoi conviventi (moglie/marito, figli che vivono in casa; badante; etc…) è stato mai consigliato il vaccino anti-influenzale? non so no sì, si sono vaccinati lo scorso autunno-inverno sì, si sono vaccinati negli ultimi 5 anni

**F6.** Secondo lei, quanto le costa effettuare la vaccinazione antinfluenzale?

□ è completamente a mie spese □ è completamente gratuita □ è parzialmente a pagamento **□** è a mie spese con rimborso parziale dalla assicurazione privata/aziendale

**□** Altro_________________________

**Sezione G. Informazioni**

Le domande in questa sezione sono mirate a valutare le fonti dalle quali acquisisce informazioni riguardo all’influenza e alla vaccinazione antinfluenzale .

**G1.** Ha avuto informazioni sulla vaccinazione antinfluenzale? no si, da chi?____________________

**G2.** Come considera la qualità delle informazioni ricevute?

insufficiente scarso buono ottimo eccellente

**G3.** Ritiene di aver bisogno di ulteriori informazioni in tema di vaccinazione antinfluenzale**?**  no si
